# A preliminary report on the feasibility of regression-based alignment of diagnostic thresholds for harmonized use of international classification criteria for antiphospholipid syndrome

**DOI:** 10.1371/journal.pone.0328229

**Published:** 2025-07-24

**Authors:** Yukari Motoki, Risa Kaneshige, Yuichiro Fujieda, Kenji Oku, Eriko Morishita, Masahiro Ieko, Tatsuya Atsumi, Kiyoshi Ichihara, Junzo Nojima

**Affiliations:** 1 Department of Laboratory Science, Faculty of Health Science, Yamaguchi University Graduate School of Medicine, Ube, Yamaguchi, Japan; 2 Department of Rheumatology, Endocrinology and Nephrology, Faculty of Medicine and Graduate School of Medicine, Hokkaido University, Sapporo, Hokkaido, Japan; 3 Division of Rheumatology, Department of Internal Medicine, Tokai University School of Medicine, Isehara, Kanagawa, Japan; 4 Department of Clinical Laboratory Sciences, Institute of Medical, Pharmaceutical and Health Sciences, Faculty of Health Sciences, Kanazawa University, Kanazawa, Ishikawa, Japan; 5 Department of Nursing, Faculty of Health and Medical Sciences, Sapporo University of Health Sciences, Sapporo, Hokkaido, Japan; Nippon Medical School, JAPAN

## Abstract

**Background:**

In 2023, international rheumatology societies issued new classification criteria for antiphospholipid syndrome (APS). The criteria require scoring antiphospholipid antibody (aPL) titers as moderate or high using the traditional thresholds of 40U or 80U determined by “standardized” ELISA. With current popularity of non-standardized aPL assays (non-ELISA), we aimed to broaden the application of the criteria by estimating equivalent thresholds for them.

**Methods:**

Four types of aPLs (aCL/aβ_2_GPI-IgG/IgM) were measured using six reagents in 50 APS and 50 non-APS patients. By regression analysis of measurements between standardized ELISA and non-ELISA assays, thresholds equivalent to 10, 20, 40 and 80U were estimated for each assay. Data points below the detection limit of each assay were excluded from the regression. The diagnostic thresholds were also evaluated using “specificity-based” method described by the International Society on Thrombosis and Haemostasis (ISTH). This approach allegedly estimates diagnostic thresholds that attain predefined specificities of 0.975 and 0.995 in distinguishing APS from non-APS cases, respectively corresponding to moderate and high titers. The between-assay concordance of diagnostic classification using the estimated thresholds was calculated as kappa coefficient (κ).

**Results:**

Using major-axis regression, thresholds equivalent to the traditional units (10 − 80U) were estimated for non-ELISA assays, which led to harmonized semi-quantitative classification with high κ values. Conversely, the specificity-based method yielded thresholds that dissociated from the traditional ones, particularly for IgG-isotype assays, resulting in lower κ values than regression-based method (P = 0.0039 − 0.0098).

**Conclusion:**

The regression-based conversion of diagnostic thresholds is practical in harmonizing diagnostic classification across major aPL assays. The specificity-based method may need adjusted predefined-specificities to estimate thresholds that are equivalent to the traditional thresholds.

## Introduction

Antiphospholipid syndrome (APS) is an autoimmune disease characterized by arterial and/or venous thrombosis and recurrent fetal loss, which is caused by antiphospholipid antibodies (aPLs) [1]. Detection of aPLs themselves or their binding activity is essential for the diagnosis of APS. Specifically, the revised Sapporo APS classification criteria issued in 2006 required positive lupus anticoagulant (LAC) test and/or the presence of aPLs (anti-cardiolipin antibodies (aCL) or anti-β_2_-glycoprotein I antibodies (aβ_2_GPI) [of IgG or IgM isotype]) with titers exceeding either the 99^th^ percentile of healthy controls or a moderate level of 40 in the traditional unit of so-called “GPL/MPL” [2].

In 2023, the new classification criteria of APS were published jointly by American College of Rheumatology (ACR) and European Alliance of Associations for Rheumatology (EULAR) called 2023 ACR/EULAR APS classification criteria. As laboratory criteria of APS, it proposed to determine a weighted cumulative score of aPLs based on the detection of LAC or semi-quantitative levels of aCL or aβ_2_GPI IgG/IgM titer, rather than the threshold of the 99^th^ percentile of healthy controls [3]. The new criteria stipulated that test results are to be obtained by the “standardized” enzyme-linked immunosorbent assay (ELISA) and expressed semi-quantitatively as moderate and high level, which respectively corresponds to 40 − 79 U and ≥80U in the traditional unit. Practically, however, popular aPL assays currently in use are performed on automated assay platforms (non-ELISA assays). This trend is attributable to their capability of simultaneous measurement of the four antibodies (aCL and aβ_2_GPI IgG/IgM) in high speed and precision. Nevertheless, they are not considered as standardized ELISA, and, therefore, are not recommended with the following note: *“if no options exist beside the use of automated-platform results for APS research, researchers should direct efforts to identifying and validating moderate/high thresholds of their platform, correlating it with aCL/anti-β*_*2*_*GPI ELISA moderate/high thresholds.*” [3].

In a recent report [4], the aPL titers that correspond to the traditional 40U and 80U thresholds measured by standardized ELISA differed greatly from titers measured by other non-ELISA assays. The Subcommittee on LA/aPLs within the Scientific and Standardization Committee (SSC) of the International Society on Thrombosis and Haemostasis (ISTH) has reported that estimation of thresholds for the semi-quantitative interpretation of aCL and aβ_2_GPI targeting 1 ELISA and 3 non-ELISA assays [5]. They employed well defined cases of 387 APS patients and 727 controls, and newly estimated diagnostic thresholds for each assay, primarily using so-called “specificity-based” method that explores thresholds that attain prespecified specificities of 0.975 and 0.995, which were allegedly meant to give moderate and high titers. However, the kappa coefficients (κ), which represent the between-assay agreement of classified results, are generally low to achieve harmonized semiquantitative classifications across the reagents.

Meanwhile, in 2022, we conducted a comprehensive method-comparison study of aPL test results targeting 5 major commercially available aPL assays [6] by measurements of 100 sera prepared from 50 each of patients with APS and non-APS. Thereby, diagnostic cutoff values that optimally distinguish the two groups were determined for each aPL assay based on the receiver-operating characteristic (ROC) analysis. We also determined each assay’s threshold aPL titer that corresponds to the 99^th^ percentile of healthy controls, as required in the revised Sapporo APS classification criteria.

Now that the 2023 ACR/EULAR APS classification criteria were issued, we found it urgently necessary to re-analyze our test results of the 5 aPL assays to estimate thresholds of each assay that are equivalent to the traditional 40 and 80U, which are essential to apply the new classification criteria. To achieve the goal, we primarily applied a linear regression analysis to convert diagnostic thresholds measured by the standardized ELISA to those of non-ELISA assays. This “regression-based” method is expected to delineate continuous linear relationships between them for the entire assay range. In this scheme, we regarded the following 2 ELISAs as the “standardized” assay: MESACUP^™^-2-test-cardiolipin (with calibrators traceable to the Harris standard [7]) and QUANTA-Lite^®^-β_2_GPI (widely used ELISA for aβ_2_GPI) assays. While we included the following aPL non-ELISA assays, all on automated platforms, as “non-standardized” assays: “QUANTA-Flash^®^” (chemiluminescent immunoassay, CLIA), “EliA^™^” (fluorescence enzyme immunoassay, FEIA), “MEBLux^™^-test” (chemiluminescent enzyme immunoassay, CLEIA), or “BioPlex^®^” (multiplex flow immunoassay, MFI).

For assessing the appropriateness of our approach, we also estimated the diagnostic thresholds by using the above-mentioned specificity-based method despite the limited sample size of our clinical cases. After estimating diagnostic thresholds in two ways, their utilities for harmonized use of the new classification criteria were compared by calculating the concordance of semiquantitative classification of aPL results.

## Materials and methods

### Clinical specimens used for the assessment of diagnostic performance

The serum specimens employed for evaluating the clinical utility of aPL assays were 100 well-defined clinical specimens obtained from the Rheumatology Department of Hokkaido University Hospital (Table 1). They comprised plasma specimens from 20 patients with primary APS (male/female 2/18; aged 14–70 years), 30 patients with APS secondary to SLE (6/24; 15–67), 10 SLE patients without APS (0/10; 16–56), and 40 patients suspected of collagen diseases of miscellaneous varieties but ruled out of both APS and SLE (17/23; 18–86). The prevalences of thrombotic/obstetrical events in patients’ history are as listed in the table.

**Table 1:** Demographic and clinical characteristics of APS and non-APS patients.

	APS		AID	
	PAPS	SAPS	SLE	Other
**Number**	**20**	**30**	**10**	**40**
**Age, mean ± SD**	**46 ± 14**	**44 ± 13**	**34 ± 13**	**59 ± 17**
**Sex, male/female**	**2/18**	**6/24**	**0/10**	**17/23**
**Thrombosis, n**	**14**	**24**	**0**	**0**
AT, n	9	18	0	0
VT, n	7	12	0	0
**Obstetrical complication, n**	**6**	**9**	**0**	**0**

PAPS, primary antiphospholipid syndrome; SAPS, secondary antiphospholipid syndrome; AID, autoimmune disease; SLE, systemic lupus erythematosus; AT, arterial thrombosis; VT, venous thrombosis. The breakdown numbers include duplicate incidence of complications.

The diagnosis of SLE was made based on the 1997 revised criteria of the American College of Rheumatology Criteria for Classification of Systemic Lupus Erythematosus [8], and that of APS was made according to the 2006 classification criteria for definite APS [2].

This study was reviewed and approved by the medical research ethics committee of Yamaguchi University Graduate School of Medicine, Faculty of Health Sciences (approval no. 363−1), and informed consent was obtained from all patients and control subjects. The retrospective data were accessed for research purposes on March 21, 2024. The authors did not have access to any information that could identify individual participants during or after data collection.

### Measurements of aPLs by ELISAs

#### MESACUP^™^-2-test.

The assay kit (MESACUP^™^-2-test-cardiolipin IgG/IgM) was provided by Medical & Biological Laboratories Co. (MBL, Nagoya, Japan). This assay, based on ELISA principle, was performed manually with Multiskan FC (Thermo-Fisher Scientific Inc., MA, USA). This assay was regarded as the standardized assay for aCL, whose calibrators were traceable to so-called Harris standards.

#### QUANTA-Lite^®^.

The assay kit (QUANTA-Lite^®^-ACA IgG/IgM and QUANTA-Lite^®^-β_2_GPI IgG/IgM ELISA) was provided by INOVA Diagnostics (CA, USA). This assay, based on ELISA principle, was performed manually with Multiskan FC. This assay was regarded as the standardized assay for aβ_2_GPI. The calibrators for aCL are internal standards correlated to the Sapporo standards (HCAL for IgG, EY2C9 for IgM) according to the reagent insert, but the details on calibrators for aβ_2_GPI are not disclosed by the manufacturer.

### Measurements of aPLs by non-ELISA assays

#### QUANTA-Flash^®^.

The reagents used to test the IgG or IgM-isotype of aCL and aβ_2_GPI, QUANTA-Flash^®^ (INOVA Diagnostics), were provided by IL Japan (Tokyo, Japan). They were measured on the platform of an ACL AcuStar autoanalyzer (Instrumentation Laboratory, MA, USA). The principle of the assay is CLIA. The calibrators of this assay are in-house reference materials that were made traceable to the Sapporo Standards.

#### EliA^™^.

The reagents used to test the IgG or IgM isotype of aCL and aβ_2_GPI (EliA^™^ Cardiolipin IgG/IgM, EliA^™^ β2-GlycoproteinI IgG/IgM) were provided by Thermo-Fisher Scientific Inc. (MA, USA). They were measured on the platform of a Phadia-100 autoanalyzer (Thermo Fisher Scientific Inc.). The principle of the assay is FEIA. This assay was standardized by use of the Harris standards.

#### MEBLux^™^-test.

The reagents used to test the IgG or IgM isotype of aβ_2_GPI (STACIA MEBLux^™^-test-β2GPI IgG/IgM) were provided by MBL. They were measured on the platform of a STACIA autoanalyzer (LSI Medience Corporation, Tokyo, Japan). The principle of the assay is CLEIA. The calibrators of the assay are in-house reference materials that are traceable to Sapporo Standards.

#### BioPlex^®^-APLS.

The reagents used to test the IgG or IgM isotype of aCL and aβ_2_GPI (BioPlex^®^ 2200 APLS IgG/IgM), were provided by Bio-Rad Laboratories Inc. (CA, USA). They were measured on the platform of a BioPlex^®^ 2200 system (Bio-Rad Laboratories Inc.). The principle of the assay is MFI. The source of the assay calibrator is not disclosed by the manufacturer.

All assays were conducted using reagents from a single lot number. Although the measurement periods varied between assays, each assay was performed within a short period of time. As a quality-control scheme common to the collaborating laboratory, the same lot of positive control specimens provided by the kit manufacturers was measured in each run of the assay.

### Statistical analyses

#### Conversion of traditional thresholds by linear regression analysis.

For comparing test results of 100 clinical specimens measured by the standardized assays with those measured by other assays, linear regression analysis was performed by using the reduced major-axis regression [9]. In the analysis, data points below the detection limit of each assay were excluded from the regression. The distributions of test results in all assays, after removal of less-than detection limit points, were highly skewed, but all of them were successfully transformed into gaussian distributions as confirmed by the linearity in the probability plot and by skewness and kurtosis tests. The precision of predicting value Y from test result X by a regression line formula of Y = a + b × X (a = intercept; b = slope) was evaluated based on the magnitude of CV of the slope, CV(b), which was calculated from the standard error of b, SE(b), as follows:


$$CV(b)=\frac{b}{SE(b)}\times 100$$

The SE(b) was estimated by the bootstrap method through 500 times resampling of the source data. As a threshold for allowing conversion of values by the regression line was set to CV(b)< 10% according to the theory presented by Ichihara [9] that is based on allowable analytical bias in clinical chemical measurements.

#### Specificity-based estimation of diagnostic thresholds.

Among the methods proposed by the ISTH-SSC for deriving diagnostic thresholds semi-quantitatively [5], we applied the specificity-based method by estimating the antibody titers of each assay that achieve prespecified specificities of 0.975 and 0.995, which allegedly give thresholds of moderate and high titers, respectively. ROC analysis was performed to determine these thresholds. In case where multiple thresholds exist for one specificity, their average was adopted. For the threshold of “low” titer, ISTH-SSC assigned the cutoff value specified by each manufacturer for their assay. However, cutoff values are not well defined by the manufacturers of this study, hence we assumed that the “low” threshold as corresponding to 20U and estimated the threshold that gives specificity of 0.95 as inferred from the specificities for the moderate and high titers.

It is of note that ISTH-SSC also employed so-called “sensitivity-based method”, which use prespecified sensitivities of 0.15 and 0.25 to estimate moderate and high titers. However, we decided not to apply it in this study because our preliminary analysis indicated that the method failed to yield appropriate thresholds for most aPL assays, likely due to the narrow overlap in antibody titer distributions between APS and non-APS cases for our dataset.

#### Determination of the 99^th^ percentile of test results for healthy controls.

The computation method for the 99^th^ percentile of healthy controls is as described previously [6]. In short, serum test results of the healthy volunteers measured by each assay reagent were logarithmically transformed to attain Gaussian distribution. The normality of the distribution was confirmed by Kolmogorov-Smirnov test and the linearity in the probability plot (so-called Q-Q plot). The 99^th^ percentile was calculated as exp (mean^T^ + 2.236 × SD^T^) where mean^T^ and SD^T^ represent the mean and standard deviation (SD) of test results computed under logarithmic scale. On the other hand, when Gaussian transformation failed due to data containing less than detection limit values, Gaussian transformation was performed again after the removal of those points. In case, the transformation still failed, the 99^th^ percentile was estimated by nonparametrically with adjustment using linear interpolation around the percentile.

#### Concordance of diagnostic classification using estimated thresholds.

For each aPL assay reagent, test results were classified into 4 categories: ≤ 20U, 20–40U, 40–80U, ≥ 80U. The concordance of the classification between any two aPL assays was calculated from 4 × 4 frequency matrix (so-called confusion matrix) as Cohen’s kappa coefficient (κ). We interpreted the practical significance (effect size) of κ, in accordance with the interpretations adopted in the ISTH-SCC paper [5], as follows: none to slight agreement for κ < 0.21, minimal agreement for 0.21 ≤ κ < 39, weak agreement for 0.40 ≤ κ < 0.59, moderate agreement for 0.60 ≤ κ < 0.79, strong agreement for 0.80 ≤ κ < 0.90, and almost perfect for 0.90 ≤ κ.

## Results

### Precision of converting diagnostic thresholds by linear regression

**Fig 1:**
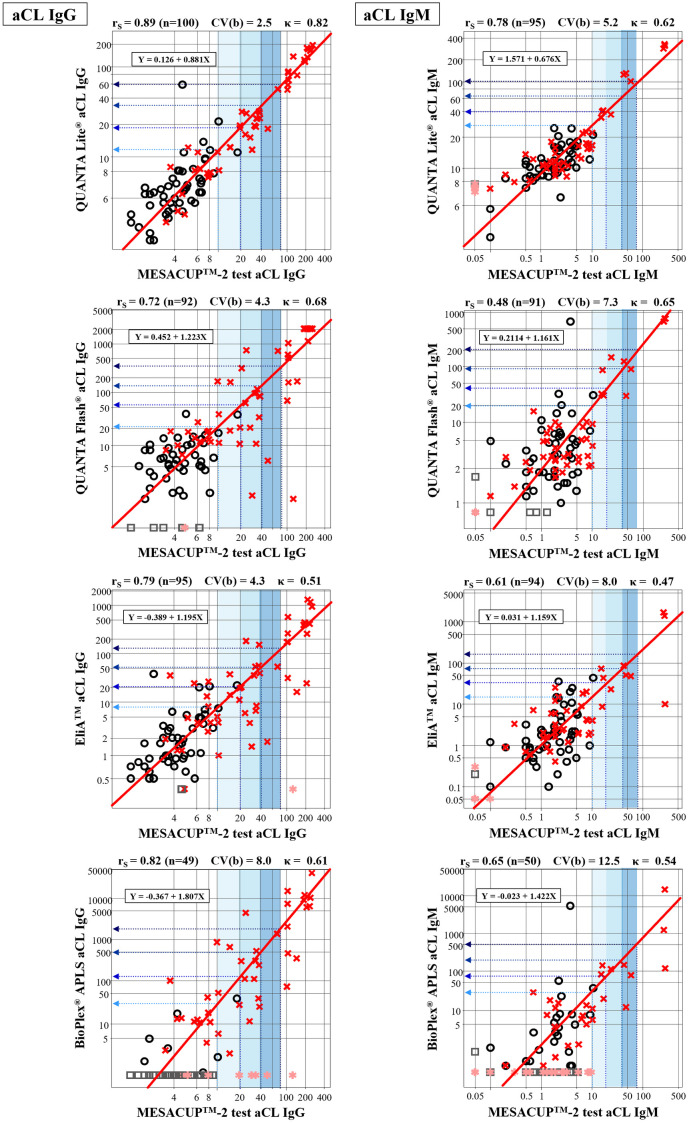
Correlations and regression lines of test results between the reference assay and other assays (aCL). The regression line was estimated by the major-axis regression. The correlation coefficient was calculated by Spearman’s nonparametric method. Test results of APS patients were plotted by red cross and those of non-APS patients plotted by black open-circle. Although not included in estimating the regression lines, test results below the detection limit in APS patients were plotted by pink asterisk, and those in non-APS patients were plotted by gray open-square. For legibility, the test ranges for 10 − 20, 20 − 40, and 40 − 80U by the reference assay are indicated by the graded blue shades. APS, antiphospholipid syndrome; aCL, anti-cardiolipin antibodies.

**Fig 2:**
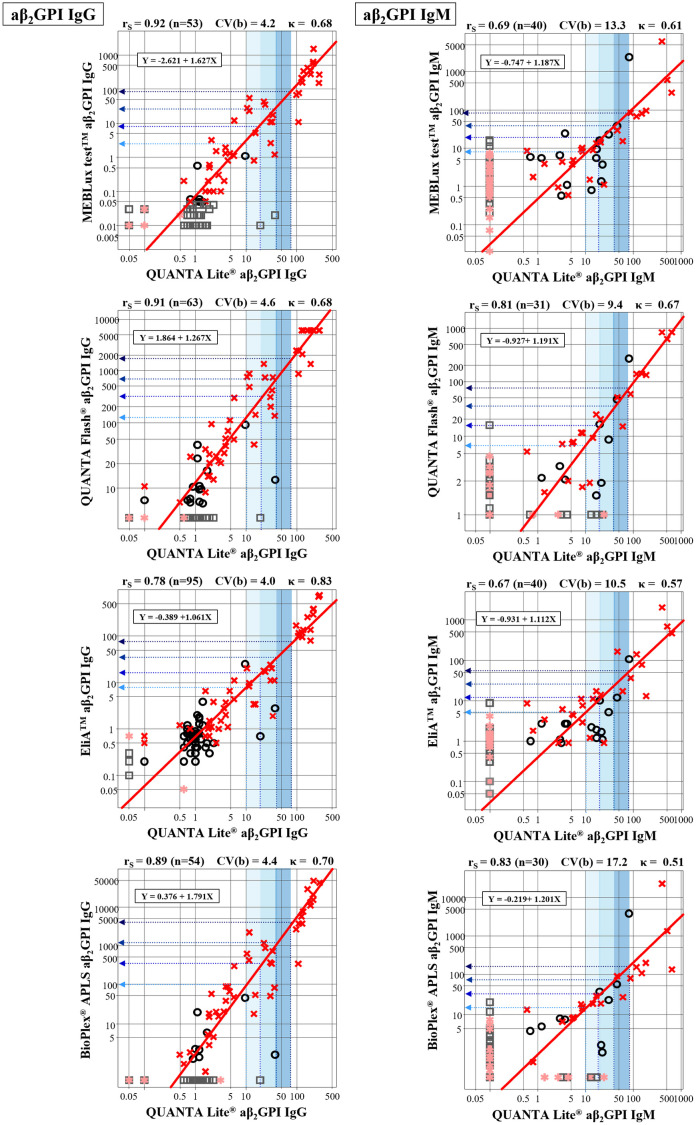
Correlations and regression lines of test results between the reference assay and other assays (aβ_2_GPI). See legend in Fig 1. APS, antiphospholipid syndrome; aβ_2_GPI, anti-β_2_-glycoprotein I antibodies.

Test results (titers) of 100 clinical cases were compared between the standardized aPL assays and other assays all pairwise, by plotting APS cases in red cross and non-APS cases in black open circle. Each relationship was presented by using the major-axis regression line and by r_S_ (Figs 1 and 2).

In aCL-IgG assays, precisions of the conversion from X to Y using the regression line were acceptable with all CV(b) less than 10% [9]. Therefore, thresholds of each assay on Y-axis that correspond to the 10U, 20U, 40U, and 80U measured by the standardized assay (MESACUP^™^-2-test-cardiolipin) were predicted by the regression line and listed in Table 2. The 90% confidence interval (CI) of prediction shown below each predicted threshold was determined by the bootstrap method from the variations of regression lines for 500 resampled results. For comparative purposes, the 99^th^ percentile and ROC-based cutoff value of each assay reagents were also listed in the table. It is of note that the equivalent thresholds for 10–80U differed greatly in QUANTA-Flash^®^ aCL, and BioPlex^®^-APLS aCL compared to the standardized assay. The 99^th^ percentiles are generally located around the level equivalent to the 10U regardless of the assays.

**Table 2:** Diagnostic thresholds determined by regression-based and specificity-based methods.

	Manu-facturer	Reagent name	Regression-based equivalent value				Specificity-based threshold			99^th^ percentile
			10U (90%CI)	20U (90%CI)	40U (90%CI)	80U (90%CI)	Low	Moderate	High	
**aCL IgG**	**MBL**	**MESACUP** ^**TM**^ **−2 test**	**10.0**	**20.0**	**40.0**	**80.0**	8.6^b^	10.1	16.3	**12.0**
	INOVA	QUANTA Lite®	11.2 (5.6–16.8)	18.6 (13.0–24.2)	32.2 (26.5–37.8)	56.9 (51.3–62.6)	10.9	20.4	58.4	7.0
	INOVA	QUANTA Flash®	20.7 (17.3–24.0)	54.3 (50.9–57.7)	134 (131–138)	324 (320–327)	19.8	34.0	36.8	24.9
	Thermo Fisher	EliA^TM^	7.8 (6.4–9.2)	21.0 (19.6–22.4)	51.9 (50.4–53.3)	123 (122–125)	20.5^c^	21.5	37.5	_—_^ a^
	Bio-Rad	BioPlex® APLS	27.4 (25.1–29.8)	123 (121–125)	485 (483–488)	1799 (1797–1802)	4.5^b^	15.1	32.3	18.5
**aCL IgM**	**MBL**	**MESACUP** ^**TM**^ **−2 test**	**10.0**	**20.0**	**40.0**	**80.0**	5.1^b^	8.9	10.4	**10.9**
	INOVA	QUANTA Lite®	27.1 (21.7–32.6)	40.8 (35.4–46.3)	62.6 (57.1–68.1)	97.5 (92.0–103)	17.9	23.9	25.3	20.4
	INOVA	QUANTA Flash®	18.6 (16.6–20.6)	40.8 (38.7–42.8)	90.3 (88.1–92.5)	201 (199–203)	15.0	31.9	681	17.8
	Thermo Fisher	EliA^TM^	14.9 (13.6–16.2)	33.2 (31.8–34.6)	74.2 (72.7–75.8)	166 (164–167)	25.0	30.5	43.5	28.1
	Bio-Rad	BioPlex® APLS	27.0 (24.4–30.0)	70.4 (67.5–73.3)	187 (183–190)	498 (494–502)	25.1	45.8	3278	25.7
**a** **β**_**2**_ **GPI IgG**	**INOVA**	**QUANTA** **Lite** **®**	**10.0**	**20.0**	**40.0**	**80.0**	4.0	17.3	37.1	**2.6**
	MBL	MEBLux^TM^ test	3.1 (1.9–4.2)	9.5 (8.3–10.7)	29.4 (28.2–30.7)	90.9 (89.6–92.2)	0.3	0.6	1.0	0.21
	INOVA	QUANTA Flash®	124 (118–130)	292 (285–298)	695 (688–701)	1665 (1658–1671)	13.1	39.0	80.9	14.4
	Thermo Fisher	EliA^TM^	7.8 (6.7–8.9)	16.3 (15.1–17.4)	33.9 (32.7–35.1)	70.8 (69.5–72.1)	3.4	3.9	24.5	3.0
	Bio-Rad	BioPlex® APLS	91.1 (88.9–93.3)	313 (310–315)	1080 (1077–1082)	3734 (3731–3736)	14.1	18.4	40.1	14.7
**a** **β**_**2**_ **GPI IgM**	**INOVA**	**QUANTA** **Lite** **®**	**10.0**	**20.0**	**40.0**	**80.0**	17.1	37.9	70.7	**18.0**
	MBL	MEBLux^TM^ test	7.3 (5.9–8.6)	16.6 (15.2–17.9)	37.7 (36.3–39.2)	85.9 (84.2–87.6)	14.7	33.3	1488	15.7
	INOVA	QUANTA Flash®	6.8 (4.8–8.9)	14.7 (12.8–16.7)	32.7 (30.7–34.8)	73.9 (71.7–76.0)	11.0	35.4	206	8.8
	Thermo Fisher	EliA^TM^	5.1 (3.8–6.4)	11.0 (9.7–12.3)	23.8 (22.4–25.2)	51.5 (50.0–53.0)	3.7	11.5	92.0	5.0
	Bio-Rad	BioPlex® APLS	14.0 (11.3–16.6)	30.6 (28.0–33.2)	68.8 (66.1–71.5)	157 (154–160)	25.5	45.7	2590	25.3

a: Excluded due to between-lot reagent mismatch during assays for determining 99^th^ percentile.

b: The specificity exceeded 97.5% when the cutoff value was set at the 99^th^ percentile, the cutoff value corresponding to 95% specificity was adopted.

c: The 99^th^ percentile could not be calculated and thus the cutoff value corresponding to 95% specificity was adopted.

aCL, anti-cardiolipin antibodies; aβ_2_GPI, anti-β_2_-glycoprotein I antibodies; CI, confidence interval.

In contrast, in aCL IgM-isotype assay, CV(b) of the regression line between MESACUP^™^-2-test-cardiolipin and BioPlex^®^-APLS exceeded the critical level of 10%, and thus the converted values were regarded imprecise and shown just as a reference.

Regarding assays for aβ_2_GPI-IgG, the precision of conversion was all regarded acceptable. In contrast, precision of conversion for aβ_2_GPI-IgM was generally low with three out of four assays showed CV(b)>10%. This was obviously attributed to the exclusion of a large fraction (60 ~ 70%) of less-than detection limit points in those assays.

It is of note that the converted thresholds equivalent to 10–80U of the standardized assay were differed greatly especially in IgG-isotype assay of QUANTA-Flash^®^ and BioPlex^®^-APLS while converted thresholds of other two IgG-isotype assays were closer to the traditional values.

### Comparison of estimated thresholds by regression-based and specificity-based methods

The thresholds estimated by the regression-based and specificity-based methods were compared in Table 2. They generally did not match well. For instance, in IgG-isotype assays, specificity-based thresholds for moderate and high levels were quite different from the thresholds equivalent to 40 and 80U estimated by regression-based method. This gap was observed not only for the standardized assay but also for other non-ELISA assays. This implies that the specificity-based threshold estimates cannot be regarded as equivalent to 40 and 80U, which are required for semiquantitative scoring of aPL test results. As for IgM-isotype assays, the discrepancies in thresholds between the two prediction methods are also mixed, and again the specificity-based threshold estimates are not directly applicable to the semi-quantitative scoring of aPL test results.

**Fig 3:**
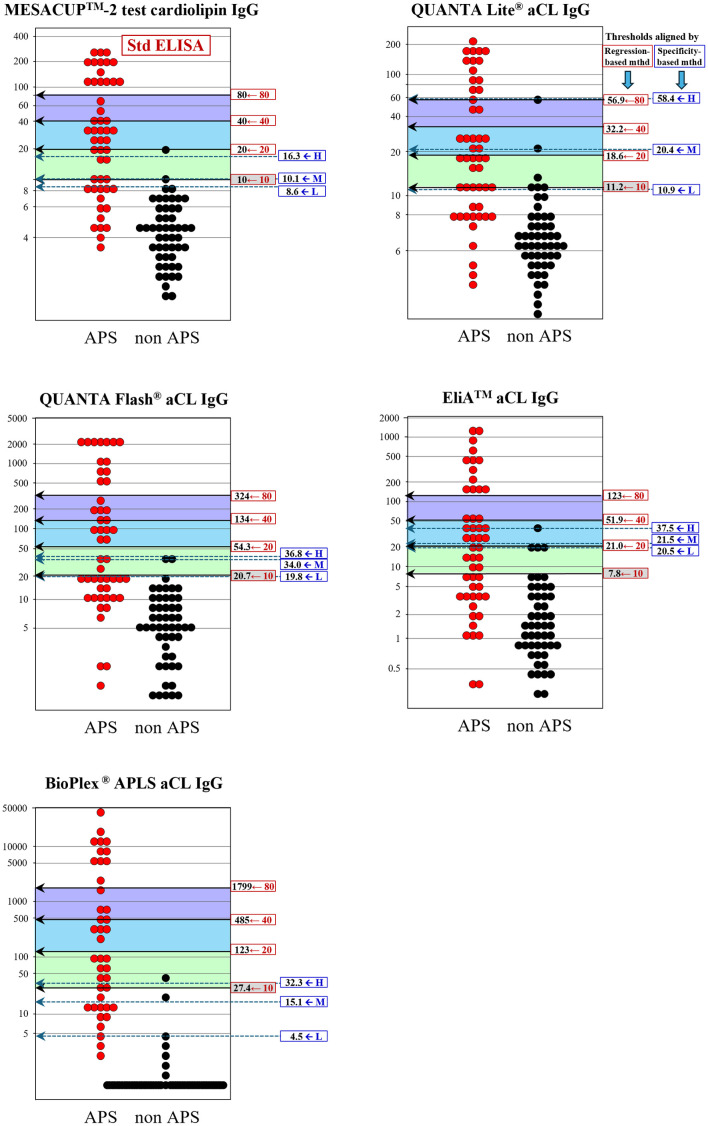
Comparison of diagnostic thresholds determined by regression-based and specificity-based methods (aCL IgG). Diagnostic thresholds of aPL assays that correspond to 10, 20, 40, and 80U of the standardized assay were determined by the regression-based method and shown in relation to the actual distributions of test results for 50 APS and 50 non-APS cases. Shown in parallel are the low, moderate, and high thresholds determined by specificity-based method using preset specificities of 0.95, 0.975, and 0.99, respectively. The threshold corresponding to 10U was only shown for the regression-based method due to unavailability of predefined specificity corresponding to 10U in the specificity-based method. APS, antiphospholipid syndrome; aCL, anti-cardiolipin antibodies; aβ_2_GPI, anti-β_2_-glycoprotein I antibodies; H, high; M, moderate; L, low.

**Fig 4:**
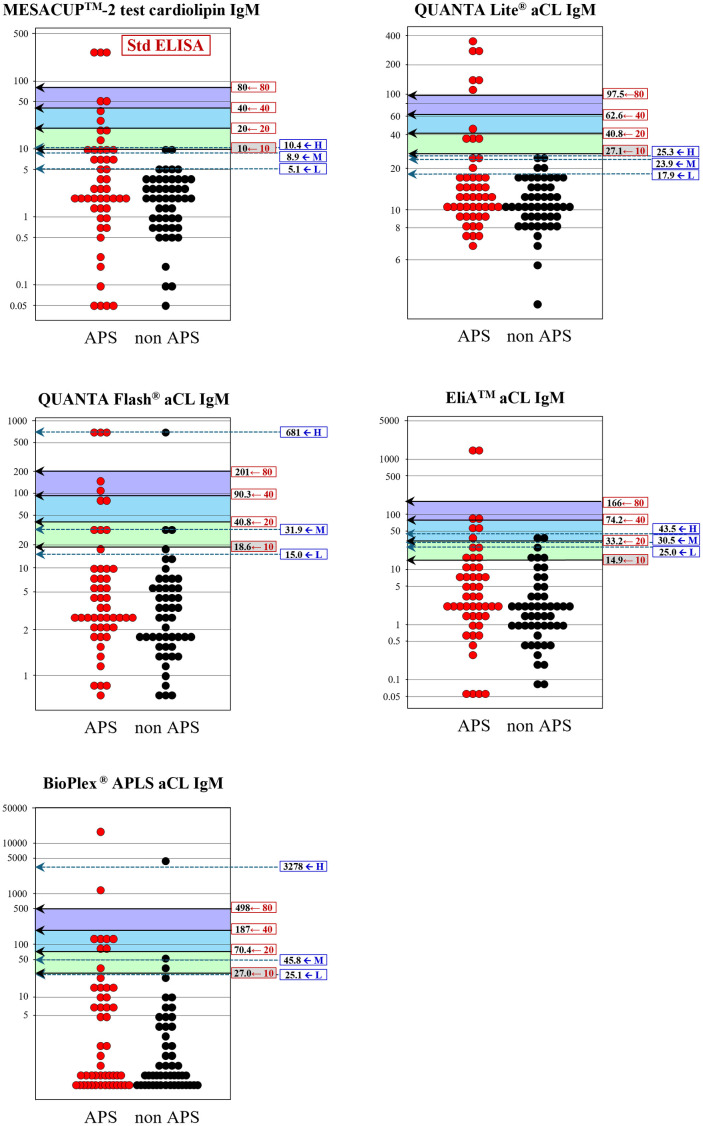
Comparison of diagnostic thresholds determined by regression-based and specificity-based methods (aCL IgM). See legend in Fig 3.

**Fig 5:**
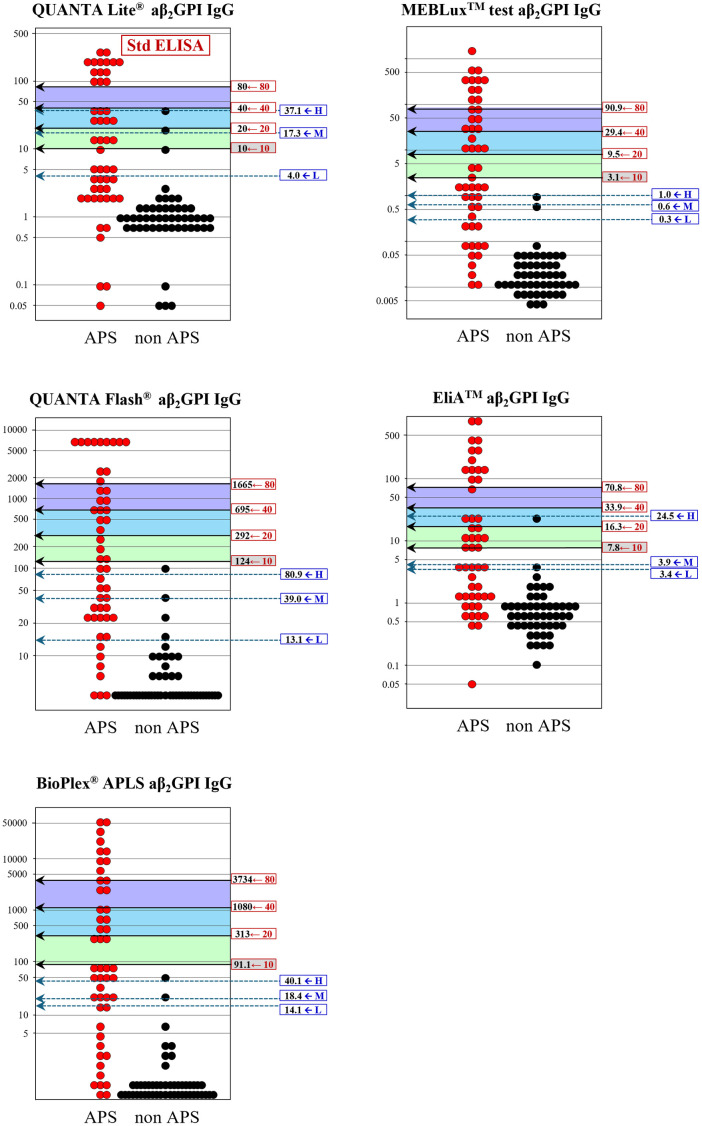
Comparison of diagnostic thresholds determined by regression-based and specificity-based methods (aβ_2_GPI IgG). See legend in Fig 3.

**Fig 6:**
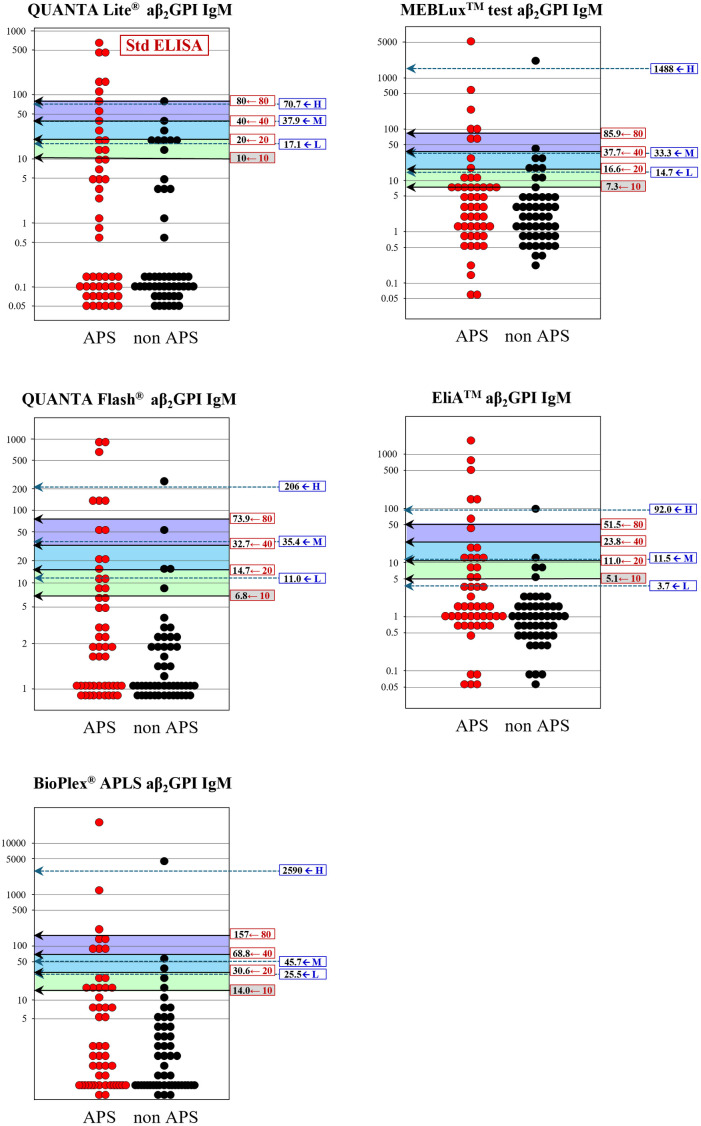
Comparison of diagnostic thresholds determined by regression-based and specificity-based methods (aβ_2_GPI IgM). See legend in Fig 3.

These problems of inconsistencies observed between the two threshold estimation methods are graphically apparent in Figs 3–6: i.e., the wide gap of the specificity-based thresholds from the traditional 40, 80U especially in IgG-isotype assays. Besides, very narrow between-threshold spacing observed in some aPL assays indicates that the specificity-based method requires a larger sample size to get better resolution of the thresholds for practical use.

### Between-assay concordance of classification using the predicted thresholds

To evaluate between-assay concordance of classification based on thresholds equivalent to 20, 40, and 80U, the confusion matrix was constructed as shown in Table 3 for pairs of assay results between the standardized assays (MESACUP^™^-2-test aCL-IgG, QUANTA-Lite^®^ aβ_2_GPI-IgG) and other assays. The κ was calculated from each matrix. The results indicate that κ is appreciably high (0.51 ≤ κ ≤ 0.83) and thus the conversion of the three thresholds from the standardized assays to the other assays is regarded as valid (Table 3, S1 Table). In contrast, the concordance of classification using regression-based thresholds for IgM-isotype assays, both for aCL and aβ_2_GPI, was generally weaker (0.41 ≤ κ ≤ 0.75), as evidenced by generally lower κ (see S2 Table). Similar confusion matrices were constructed for the thresholds predicted by the specificity-based method as presented in S3 and S4 Table.

**Table 3:** Concordance of harmonized antibody titers between reference assay and other assays.

	Estimated threshold			MESACUP^TM^-2 test aCL IgG				κ	95%CI
				< 20U	20–40U	40–80U	80U ≤		
**QUANTA Lite®** **aCL IgG**	< 20U → < 19			68	3	1	0	0.82	0.68–0.96
	20–40U → 19–32			2	9	0	0		
	40–80U → 32–57			0	0	1	1		
	80U ≤ → 57 ≤			1	0	0	14		
**QUANTA Flash** **®** **aCL IgG**	< 20U → < 54			69	4	1	1	0.68	0.55–0.82
	20–40U → 54–134			0	6	0	1		
	40–80U → 134–324			2	1	0	2		
	80U ≤ → 324 ≤			0	1	1	11		
**EliA** ^**TM**^ **aCL IgG**	< 20U → < 21			64	6	1	2	0.51	0.37–0.65
	20–40U → 21–52			7	2	0	2		
	40–80U → 52–123			0	2	1	0		
	80U ≤ → 123 ≤			0	2	0	11		
**BioPlex** **®** **aCL IgG**	< 20U → < 123			69	7	1	2	0.61	0.48–0.75
	20–40U → 123–485			0	3	0	2		
	40–80U → 485–1799			2	1	1	0		
	80U ≤ → 1799 ≤			0	1	0	11		
	**Estimated threshold**			**QUANTA Lite® aβ**_**2**_ **GPI IgG**				**κ**	**95%CI**
				**< 20U**	**20**–**40U**	**40**–**80U**	**80U ≤**		
**MEBLux**^**TM**^ **test** **aβ**_**2**_**GPI IgG**	< 20U → < 9.5			74	3	0	0	0.68	0.54–0.82
	20–40U → 9.5–29			3	3	0	1		
	40–80U → 29–91			1	2	0	2		
	80U ≤ → 91 ≤			0	0	0	11		
**QUANTA Flash®** **aβ**_**2**_ **GPI IgG**	< 20U → < 292			74	3	0	0	0.68	0.55–0.82
	20–40U → 292–695			2	2	0	0		
	40–80U → 695–1665			2	3	0	2		
	80U ≤ → 1665 ≤			0	0	0	12		
**EliA** ^**TM**^ **aβ**_**2**_ **GPI IgG**	< 20U → < 16			76	4	0	0	0.83	0.67–0.98
	20–40U → 16–34			2	4	0	0		
	40–80U → 34–71			0	0	0	0		
	80U ≤ → 71 ≤			0	0	0	14		
**BioPlex** **®** **aβ**_**2**_ **GPI IgG**	< 20U → < 313			75	3	0	0	0.70	0.57–0.84
	20–40U → 313–1080			2	4	0	1		
	40–80U → 1080–3734			1	1	0	3		
	80 ≤ → 3734 ≤			0	0	0	10		

aCL, anti-cardiolipin antibodies; aβ_2_GPI, anti-β_2_-glycoprotein I antibodies; CI, confidence interval.

As a whole, the degree of harmonized classification across all assays was significantly higher by using regression-based thresholds in all four types of aPL assays when tested by Wilcoxon’s signed rank test with P-values ranging from 0.0039 − 0.0098 (Table 4).

**Table 4:** Concordance of semiquantitative classification among aPL assays by use of harmonized thresholds.

	(1) Regression-based				(2) Specificity-based				(1) vs (2) P value
aCL-IgG	QUANTA Lite®	QUANTA Flash®	EliA^TM^	BioPlex®	QUANTA Lite®	QUANTA Flash®	EliA^TM^	BioPlex®	0.0039
MESACUP^TM^	0.82	0.68	0.51	0.61	0.66	0.49	0.47	0.53	
QUANTALite®	―	0.65	0.50	0.63	―	0.48	0.33	0.35	
QUANTAFlash®	―	―	0.51	0.77	―	―	0.54	0.68	
EliA^TM^	―	―	―	0.48	―	―	―	0.42	
aCL-IgM	QUANTA Lite®	QUANTA Flash®	EliA^TM^	BioPlex®	QUANTA Lite®	QUANTA Flash®	EliA^TM^	BioPlex®	0.0059
MESACUP^TM^	0.62	0.65	0.47	0.54	0.61	0.24	0.36	0.20	
QUANTALite®	―	0.51	0.54	0.41	―	0.36	0.46	0.31	
QUANTAFlash®	―	―	0.44	0.75	―	―	0.29	0.80	
EliA^TM^	―	―	―	0.46	―	―	―	0.25	
aβ_2_GPI IgG	MEBLux^TM^	QUANTA Flash®	EliA^TM^	BioPlex®	MEBLux^TM^	QUANTA Flash®	EliA^TM^	BioPlex®	0.0098
QUANTALite®	0.68	0.68	0.83	0.70	0.51	0.43	0.72	0.51	
MEBLux^TM^	―	0.79	0.73	0.92	―	0.77	0.50	0.89	
QUANTAFlash®	―	―	0.73	0.71	―	―	0.44	0.76	
EliA^TM^	―	―	―	0.75	―	―	―	0.50	
aβ_2_GPI IgM	MEBLux^TM^	QUANTA Flash®	EliA^TM^	BioPlex®	MEBLux^TM^	QUANTA Flash®	EliA^TM^	BioPlex®	0.1768
QUANTALite®	0.61	0.67	0.57	0.51	0.49	0.55	0.48	0.50	
MEBLux^TM^	―	0.62	0.51	0.72	―	0.61	0.47	0.80	
QUANTAFlash®	―	―	0.69	0.69	―	―	0.66	0.78	
EliA^TM^	―	―	―	0.54	―	―	―	0.48	

pale orange, 0.21 ≤ κ < 0.39 (minimal agreement); light orange, 0.40 ≤ κ < 0.59 (weak agreement); medium orange, 0.60 ≤ κ < 0.79 (moderate agreement); deep orange, 0.80 ≤ κ < 0.90 (strong agreement); bright orange, 0.90 ≤ κ (almost perfect agreement).

aCL, anti-cardiolipin antibodies; aβ_2_GPI, anti-β_2_-glycoprotein I antibodies.

## Discussion

As a limitation common to immunoassays for autoantibodies with extreme heterogeneity in assay reagents, aPL assays of all kinds exhibit large differences in measured values. To ameliorate the situation, Harris et al. prepared reference materials of IgG and IgM fractions known as “Harris standards” from sera of representative APS patients [7,10]. They were value assigned in reporting units of GPL and MPL, respectively representing binding activities of purified IgG and IgM [7]. The units have been used since then in calibrators of some ELISA, and in secondary calibrators and monoclonal antibodies [11–14], which were subsequently made traceable to the Harris standards. Thus, the standards played an important role in the harmonized reporting of the aPL results. However, with persistence of large between-assay differences in test results, Harris *et al.* proposed semi-quantitative reporting of aPL antibody titers as low, moderate, or high [7,15,16], which are in recent years regarded as corresponding to <40U, 40 − 79U, 80U≤ , respectively, as measured using the Harris standards.

This semiquantitative interpretation was adopted for diagnostic classification of aPL test results in the latest classification criteria issued in 2023 by ACR/EULAR, which made it obligatory to determine the traditional diagnostic thresholds of 40 and 80U as measured by the “standardized” ELISA in calculating cumulative scores of aPL test results for using APS classification criteria. Nevertheless, a majority of aPL assays currently performed on non-ELISA are not “standardized”. Hence, it became urgently necessary to transfer the specified diagnostic thresholds from “standardized” ELISA to all other aPL assays to promote the use of the new classification criteria.

To achieve the goal, we resorted to the major-axis regression analysis to estimate the aPL titers equivalent to the traditional 20, 40 and 80U by deriving all pairwise regression lines between test results of standardized ELISA and non-ELISA assays for four types of aPL assays (aCL/aβ_2_GPI IgG/IgM). Based on our criteria of reliability in linear conversion of CV(b)<10%, which represents coefficient of variation computed for the standard-error of slope (b) or the tightness of the scatter around the regression line [9], we regarded that the conversions were successful almost unanimously for IgG-isotype assays as shown in Table 2 listing the converted thresholds together with their 90% CI. In contrast, for IgM-isotype assays, the CV(b) of conversion often exceeded 10% due to small data points remaining after the exclusion of less-than detection limit values. Therefore, we must regard their converted values presumptive. Nevertheless, the linearity of the regression line was clearly observed with relatively narrow 90% confidence intervals of the predicted thresholds.

By using the three thresholds (20, 40, 80) converted for all non-ELISA assays, the concordances in classification of 100 clinical cases were κ = 0.48 ~ 0.82 among five aCL-IgG assays and 0.68 ~ 0.92 among five aβ_2_GPI-IgG assays as shown in Table 4.

For assessment of appropriateness of our results, we also determined the thresholds by using the “specificity-based” method presented by ISTH, despite the limitation of much smaller size of our clinical cases to obtain reliable results. By using prespecified specificities of 0.975 and 0.99, which allegedly give thresholds distinguishing moderate and high titers. For the threshold to distinguish low level, ISTH adopted manufacturer’s cutoff values of each assay, however, due to unavailability of the cutoff values in some assays, we rather determined the low threshold that gave the specificity of 0.95 in analogy to the other thresholds. Direct comparison of thresholds derived by the two methods revealed unexpectedly large discrepancies. The notable problem of the specificity-based threshold estimates for moderate and high was their mismatch from the traditional 40 and 80U, which are requested for use in semiquantitative scoring in the new classification criteria, as evidently shown in Table 2 and Figs 3–6. Importantly, even for the IgG isotype that plays a key role in laboratory criteria for APS, the thresholds estimated by the specificity-based method using ELISA assays were deviated appreciably from the standardized values of 40 and 80 U required in the criteria. This discrepancy raises some concerns about the suitability of specificity-based thresholds for classifying IgG aPL levels as requested in the current APS classification criteria. Moreover, the specificity-based method is highly dependent on the sample population, and even when using the same specificity level, thresholds can vary depending on the composition of cohorts. Besides, a proper specification of predefined specificities is essential. Narrow between-threshold spacings in IgM-isotype assay was another issue for using the specificity-based threshold estimation. In light of these findings, we consider that the specificity-based method is not appropriate for setting semiquantitative thresholds, at least for our samples of limited data size.

Besides, the concordances of the specificity-based estimation of thresholds calculated in all pairwise combinations across the aPL assays were significantly lower: κ of 0.33 ~ 0.68 for aCL-IgG and 0.43 ~ 0.89 for aβ_2_GPI-IgG. The results imply that, although the ISTH’s specificity-based alignment of diagnostic threshold may be useful in harmonizing the semi-qualitative classification, it may not be valid for promoting the use of the new-classification criteria because estimated thresholds cannot be regarded as equivalent to the traditional thresholds of 40 and 80U. In contrast, regression-based estimation of traditional 40 and 80U is more suitable for expanding the use of the new criteria. Besides, it should be emphasized that an additional merit of our “regression-based” estimation of diagnostic thresholds is its applicability to a wide assay range and its feasibility of achieving true harmonization by recalibrating each aPL assay’s calibrator based on all-pair-wise regression equations.

### Limitation

The number of clinical samples from 50 APS and 50 non-APS patients was relatively small to convert diagnostic thresholds in high precision, especially for aPL assays of BioPlex^®^ APLS aCL IgG/IgM and QUANTA Lite^®^ aβ_2_GPI IgM, in which a large proportion of samples had values below detection limits. Therefore, we should confirm our findings by inclusion of a larger number of clinical cases, especially those with obstetric complications. Finally, the converted thresholds calculated in this study may be altered due to changes in reagent lots. Ensuring a consistent supply of the traceable calibrators is necessary to maintain harmonized thresholds across all aPL assays in the long run.

## Conclusion

Using the major-axis regression analysis, we demonstrated that it was possible to estimate diagnostic thresholds as equivalent to those measured by the standardized ELISA for non-ELISA assays. Hence, we confirmed the feasibility of applying the 2023 laboratory criteria of APS even by antibody titers measured by non-ELISA assays. Our results suggest that a true harmonization of aPL test results may be possible by using the regression lines to recalibrate the calibrators by consensus among the reagent manufacturers.

## Supporting information

S1 Table Concordance of semi-quantitative classification across all assays for IgG isotypes, using thresholds predicted by the regression-based method. aCL, anti-cardiolipin antibodies; aβ_2_GPI, anti-β_2_-glycoprotein I antibodies; CI, confidence interval.
(DOCX)

S2 Table Concordance of semi-quantitative classification across all assays for IgM isotypes, using thresholds predicted by the regression-based method. aCL, anti-cardiolipin antibodies; aβ_2_GPI, anti-β_2_-glycoprotein I antibodies; CI, confidence interval.
(DOCX)

S3 Table Concordance of semi-quantitative classification across all assays for IgG isotypes, using thresholds predicted by the specificity-based method. aCL, anti-cardiolipin antibodies; aβ_2_GPI, anti-β_2_-glycoprotein I antibodies; CI, confidence interval.
(DOCX)

S4 Table Concordance of semi-quantitative classification across all assays for IgM isotypes, using thresholds predicted by the specificity-based method. aCL, anti-cardiolipin antibodies; aβ_2_GPI, anti-β_2_-glycoprotein I antibodies; CI, confidence interval.
(DOCX)

S5 Table Raw data for antiphospholipid antibody measurements. (XLSX)
