# Supplementary material for: A preliminary report on the feasibility of regression-based alignment of diagnostic thresholds for harmonized use of international classification criteria for antiphospholipid syndrome
Source: PLoS One. 2025 Jul 24;20(7):e0328229. doi: 10.1371/journal.pone.0328229 (PMC12289022; doi:10.1371/journal.pone.0328229)
Supplement: S1 Table — aCL, anti-cardiolipin antibodies; aβ2GPI, anti-β2-glycoprotein I antibodies; CI, confidence interval. (DOCX) [file pone.0328229.s001.docx]

**S1 Table.** **Concordance of semi-quantitative classification across all assays for IgG isotypes, using thresholds predicted by the regression-based method.**

| MESACUP^TM^-2 test aCL IgG | | < 20U | 20－40U | 40－80U | 80U ≤ | kappa statistic | 95%CI |
| --- | --- | --- | --- | --- | --- | --- | --- |
| QUANTA Lite^®^  aCL IgG | < 18.6 | 68 | 3 | 1 | 0 | 0.82 | 0.682－0.964 |
|  | 18.6－32.2 | 2 | 9 | 0 | 0 |  |  |
|  | 32.2－56.9 | 0 | 0 | 1 | 1 |  |  |
|  | 56.9 ≤ | 1 | 0 | 0 | 14 |  |  |
| QUANTA Flash^®^  aCL IgG | < 54.3 | 69 | 4 | 1 | 1 | 0.68 | 0.545－0.816 |
|  | 54.3－134.4 | 0 | 6 | 0 | 1 |  |  |
|  | 134.4－323.5 | 2 | 1 | 0 | 2 |  |  |
|  | 323.5 ≤ | 0 | 1 | 1 | 11 |  |  |
| EliA^TM^  aCL IgG | < 21.0 | 64 | 6 | 1 | 2 | 0.51 | 0.370－0.648 |
|  | 21.0－51.9 | 7 | 2 | 0 | 2 |  |  |
|  | 51.9－123.1 | 0 | 2 | 1 | 0 |  |  |
|  | 123.1 ≤ | 0 | 2 | 0 | 11 |  |  |
| BioPlex^®^  aCL IgG | < 123.1 | 69 | 7 | 1 | 2 | 0.61 | 0.479－0.749 |
|  | 123.1－485.2 | 0 | 3 | 0 | 2 |  |  |
|  | 485.2－1799.1 | 2 | 1 | 1 | 0 |  |  |
|  | 1799.1 ≤ | 0 | 1 | 0 | 11 |  |  |
| QUANTA Lite^®^ aCL IgG | | < 18.6 | 18.6－32.2 | 32.2－56.9 | 56.9 ≤ | kappa statistic | 95%CI |
| QUANTA Flash^®^  aCL IgG | < 54.3 | 69 | 4 | 0 | 2 | 0.65 | 0.516－0.789 |
|  | 54.3－134.4 | 1 | 5 | 1 | 0 |  |  |
|  | 134.4－323.5 | 2 | 1 | 0 | 2 |  |  |
|  | 323.5 ≤ | 0 | 1 | 1 | 11 |  |  |
| EliA^TM^  aCL IgG | < 21.0 | 65 | 5 | 0 | 3 | 0.50 | 0.363－0.642 |
|  | 21.0－51.9 | 7 | 2 | 0 | 2 |  |  |
|  | 51.9－123.1 | 0 | 2 | 1 | 0 |  |  |
|  | 123.1 ≤ | 0 | 2 | 1 | 10 |  |  |
| BioPlex^®^  aCL IgG | < 123.1 | 70 | 6 | 1 | 2 | 0.63 | 0.495－0.768 |
|  | 123.1－485.2 | 0 | 3 | 0 | 2 |  |  |
|  | 485.2－1799.1 | 2 | 1 | 1 | 0 |  |  |
|  | 1799.1 ≤ | 0 | 1 | 0 | 11 |  |  |
| QUANTA Flash^®^ aCL IgG | | < 54.3 | 54.3－134.4 | 134.4－323.5 | 323.5 ≤ | kappa statistic | 95%CI |
| EliA^TM^  aCL IgG | < 21.0 | 67 | 2 | 4 | 0 | 0.51 | 0.372－0.643 |
|  | 21.0－51.9 | 7 | 2 | 0 | 2 |  |  |
|  | 51.9－123.1 | 1 | 1 | 0 | 1 |  |  |
|  | 123.1 ≤ | 0 | 2 | 1 | 10 |  |  |
| BioPlex^®^  aCL IgG | < 123.1 | 75 | 4 | 0 | 0 | 0.77 | 0.630－0.904 |
|  | 123.1－485.2 | 0 | 2 | 3 | 0 |  |  |
|  | 485.2－1799.1 | 0 | 1 | 2 | 1 |  |  |
|  | 1799.1 ≤ | 0 | 0 | 0 | 12 |  |  |
| EliA^TM^ aCL IgG | | < 21.0 | 21.0－51.9 | 51.9－123.1 | 123.1 ≤ | kappa statistic | 95%CI |
| BioPlex^®^  aCL IgG | < 123.1 | 68 | 9 | 1 | 1 | 0.48 | 0.341－0.611 |
|  | 123.1－485.2 | 2 | 0 | 1 | 2 |  |  |
|  | 485.2－1799.1 | 3 | 0 | 1 | 0 |  |  |
|  | 1799.1 ≤ | 0 | 2 | 0 | 10 |  |  |

| QUANTA Lite^®^ aβ_2_GPI IgG | | < 20U | 20－40U | 40－80U | 80U ≤ | kappa statistic | 95%CI |
| --- | --- | --- | --- | --- | --- | --- | --- |
| MEBLux^TM^ test  aβ_2_GPI IgG | < 9.5 | 74 | 3 | 0 | 0 | 0.68 | 0.544－0.822 |
|  | 9.5－29.4 | 3 | 3 | 0 | 1 |  |  |
|  | 29.4－90.9 | 1 | 2 | 0 | 2 |  |  |
|  | 90.9 ≤ | 0 | 0 | 0 | 11 |  |  |
| QUANTA Flash^®^  aβ_2_GPI IgG | < 291.6 | 74 | 3 | 0 | 0 | 0.68 | 0.547－0.820 |
|  | 291.6－694.7 | 2 | 2 | 0 | 0 |  |  |
|  | 694.7－1664.5 | 2 | 3 | 0 | 2 |  |  |
|  | 1664.5 ≤ | 0 | 0 | 0 | 12 |  |  |
| EliA^TM^  aβ_2_GPI IgG | < 16.3 | 76 | 4 | 0 | 0 | 0.83 | 0.675－0.984 |
|  | 16.3－33.9 | 2 | 4 | 0 | 0 |  |  |
|  | 33.9－70.8 | 0 | 0 | 0 | 0 |  |  |
|  | 70.8 ≤ | 0 | 0 | 0 | 14 |  |  |
| BioPlex^®^  aβ_2_GPI IgG | < 312.7 | 75 | 3 | 0 | 0 | 0.70 | 0.566－0.843 |
|  | 312.7－1079.6 | 2 | 4 | 0 | 1 |  |  |
|  | 1079.6－3733.7 | 1 | 1 | 0 | 3 |  |  |
|  | 3733.7 ≤ | 0 | 0 | 0 | 10 |  |  |
| MEBLux^TM^ test aβ_2_GPI IgG | | < 9.5 | 9.5－29.4 | 29.4－90.9 | 90.9 ≤ | kappa statistic | 95%CI |
| QUANTA Flash^®^  aβ_2_GPI IgG | < 291.6 | 77 | 0 | 0 | 0 | 0.79 | 0.659－0.928 |
|  | 291.6－694.7 | 0 | 3 | 1 | 0 |  |  |
|  | 694.7－1664.5 | 0 | 4 | 2 | 1 |  |  |
|  | 1664.5 ≤ | 0 | 0 | 2 | 10 |  |  |
| EliA^TM^  aβ_2_GPI IgG | < 16.3 | 76 | 3 | 1 | 0 | 0.73 | 0.585－0.866 |
|  | 16.3－33.9 | 1 | 3 | 2 | 0 |  |  |
|  | 33.9－70.8 | 0 | 0 | 0 | 0 |  |  |
|  | 70.8 ≤ | 0 | 1 | 2 | 11 |  |  |
| BioPlex^®^  aβ_2_GPI IgG | < 312.7 | 77 | 1 | 0 | 0 | 0.92 | 0.787－1.000 |
|  | 312.7－1079.6 | 0 | 6 | 1 | 0 |  |  |
|  | 1079.6－3733.7 | 0 | 0 | 4 | 1 |  |  |
|  | 3733.7 ≤ | 0 | 0 | 0 | 10 |  |  |
| QUANTA Flash^®^ aβ_2_GPI IgG | | < 291.6 | 291.6－694.7 | 694.7－1664.5 | 1664.5 ≤ | kappa statistic | 95%CI |
| EliA^TM^  aβ_2_GPI IgG | < 16.3 | 76 | 2 | 2 | 0 | 0.73 | 0.587－0.865 |
|  | 16.3－33.9 | 1 | 2 | 3 | 0 |  |  |
|  | 33.9－70.8 | 0 | 0 | 0 | 0 |  |  |
|  | 70.8 ≤ | 0 | 0 | 2 | 12 |  |  |
| BioPlex^®^  aβ_2_GPI IgG | < 312.7 | 77 | 1 | 0 | 0 | 0.71 | 0.578－0.845 |
|  | 312.7－1079.6 | 0 | 2 | 5 | 0 |  |  |
|  | 1079.6－3733.7 | 0 | 1 | 1 | 3 |  |  |
|  | 3733.7 ≤ | 0 | 0 | 1 | 9 |  |  |
| EliA^TM^ aβ_2_GPI IgG | | < 16.3 | 16.3－33.9 | 33.9－70.8 | 70.8 ≤ | kappa statistic | 95%CI |
| BioPlex^®^  aβ_2_GPI IgG | < 312.7 | 77 | 1 | 0 | 0 | 0.75 | 0.609－0.888 |
|  | 312.7－1079.6 | 2 | 4 | 0 | 1 |  |  |
|  | 1079.6－3733.7 | 1 | 1 | 0 | 3 |  |  |
|  | 3733.7 ≤ | 0 | 0 | 0 | 10 |  |  |

aCL, anti-cardiolipin antibody; aβ_2_GPI, anti-β_2_-glycoprotein I antibody; CI, confidence interval
