# Supplementary material for: A preliminary report on the feasibility of regression-based alignment of diagnostic thresholds for harmonized use of international classification criteria for antiphospholipid syndrome
Source: PLoS One. 2025 Jul 24;20(7):e0328229. doi: 10.1371/journal.pone.0328229 (PMC12289022; doi:10.1371/journal.pone.0328229)
Supplement: S2 Table — aCL, anti-cardiolipin antibodies; aβ2GPI, anti-β2-glycoprotein I antibodies; CI, confidence interval. (DOCX) [file pone.0328229.s002.docx]

**S2 Table. Concordance of semi-quantitative classification across all assays for IgM isotypes, using thresholds predicted by the regression-based method.**

| MESACUP^TM^-2 test aCL IgM | | < 20 | 20－40 | 40－80 | 80 ≤ | kappa statistic | 95%CI |
| --- | --- | --- | --- | --- | --- | --- | --- |
| QUANTA Lite^®^  aCL IgM | < 40.8 | 92 | 1 | 0 | 0 | 0.62 | 0.483－0.766 |
|  | 40.8－62.6 | 1 | 0 | 0 | 0 |  |  |
|  | 62.6－97.5 | 0 | 0 | 0 | 0 |  |  |
|  | 97.5 ≤ | 0 | 0 | 3 | 3 |  |  |
| QUANTA Flash^®^  aCL IgM | < 40.8 | 91 | 0 | 1 | 0 | 0.65 | 0.511－0.787 |
|  | 40.8－90.3 | 1 | 0 | 1 | 0 |  |  |
|  | 90.3－201.1 | 0 | 1 | 1 | 0 |  |  |
|  | 201.1 ≤ | 1 | 0 | 0 | 3 |  |  |
| EliA^TM^  aCL IgM | < 33.2 | 89 | 1 | 0 | 1 | 0.47 | 0.350－0.598 |
|  | 33.2－74.2 | 4 | 0 | 2 | 0 |  |  |
|  | 74.2－165.8 | 0 | 0 | 1 | 0 |  |  |
|  | 165.8 ≤ | 0 | 0 | 0 | 2 |  |  |
| BioPlex^®^  aCL IgM | < 70.4 | 90 | 0 | 1 | 0 | 0.54 | 0.416－0.664 |
|  | 70.4－186.6 | 2 | 1 | 2 | 1 |  |  |
|  | 186.6－497.9 | 0 | 0 | 0 | 0 |  |  |
|  | 497.9 ≤ | 1 | 0 | 0 | 2 |  |  |
| QUANTA Lite^®^ aCL IgM | | < 40.8 | 40.8－62.6 | 62.6－97.5 | 97.5 ≤ | kappa statistic | 95%CI |
| QUANTA Flash^®^  aCL IgM | < 40.8 | 90 | 1 | 0 | 1 | 0.51 | 0.357－0.655 |
|  | 40.8－90.3 | 1 | 0 | 0 | 1 |  |  |
|  | 90.3－201.1 | 1 | 0 | 0 | 1 |  |  |
|  | 201.1 ≤ | 1 | 0 | 0 | 3 |  |  |
| EliA^TM^  aCL IgM | < 33.2 | 90 | 0 | 0 | 1 | 0.54 | 0.410－0.668 |
|  | 33.2－74.2 | 3 | 1 | 0 | 2 |  |  |
|  | 74.2－165.8 | 0 | 0 | 0 | 1 |  |  |
|  | 165.8 ≤ | 0 | 0 | 0 | 2 |  |  |
| BioPlex^®^  aCL IgM | < 70.4 | 89 | 1 | 0 | 1 | 0.41 | 0.266－0.545 |
|  | 70.4－186.6 | 3 | 0 | 0 | 3 |  |  |
|  | 186.6－497.9 | 0 | 0 | 0 | 0 |  |  |
|  | 497.9 ≤ | 1 | 0 | 0 | 2 |  |  |
| QUANTA Flash^®^ aCL IgM | | < 40.8 | 40.8－90.3 | 90.3－201.1 | 201.1 ≤ | kappa statistic | 95%CI |
| EliA^TM^  aCL IgM | < 33.2 | 87 | 1 | 1 | 2 | 0.44 | 0.307－0.572 |
|  | 33.2－74.2 | 5 | 1 | 0 | 0 |  |  |
|  | 74.2－165.8 | 0 | 0 | 1 | 0 |  |  |
|  | 165.8 ≤ | 0 | 0 | 0 | 2 |  |  |
| BioPlex^®^  aCL IgM | < 70.4 | 91 | 0 | 0 | 0 | 0.75 | 0.615－0.886 |
|  | 70.4－186.6 | 1 | 2 | 2 | 1 |  |  |
|  | 186.6－497.9 | 0 | 0 | 0 | 0 |  |  |
|  | 497.9 ≤ | 0 | 0 | 0 | 3 |  |  |
| EliA^TM^ aCL IgM | | < 33.2 | 33.2－74.2 | 74.2－165.8 | 165.8 ≤ | kappa statistic | 95%CI |
| BioPlex^®^  aCL IgM | < 70.4 | 87 | 4 | 0 | 0 | 0.46 | 0.309－0.618 |
|  | 70.4－186.6 | 3 | 2 | 1 | 0 |  |  |
|  | 186.6－497.9 | 0 | 0 | 0 | 0 |  |  |
|  | 497.9 ≤ | 1 | 0 | 0 | 2 |  |  |

| QUANTA Lite^®^ aβ_2_GPI IgM | | < 20 | 20－40 | 40－80 | 80 ≤ | kappa statistic | 95%CI |
| --- | --- | --- | --- | --- | --- | --- | --- |
| MEBLux^TM^ test  aβ_2_GPI IgM | < 16.6 | 83 | 4 | 1 | 0 | 0.61 | 0.473－0.740 |
|  | 16.6－37.7 | 1 | 1 | 1 | 0 |  |  |
|  | 37.7－85.9 | 0 | 0 | 1 | 3 |  |  |
|  | 85.9 ≤ | 0 | 0 | 0 | 5 |  |  |
| QUANTA Flash^®^  aβ_2_GPI IgM | < 14.7 | 81 | 4 | 1 | 0 | 0.67 | 0.527－0.804 |
|  | 14.7－32.7 | 3 | 1 | 0 | 0 |  |  |
|  | 32.7－73.9 | 0 | 0 | 2 | 1 |  |  |
|  | 73.9 ≤ | 0 | 0 | 0 | 7 |  |  |
| EliA^TM^  aβ_2_GPI IgM | < 11.0 | 81 | 4 | 0 | 0 | 0.57 | 0.427－0.706 |
|  | 11.0－23.8 | 3 | 1 | 2 | 1 |  |  |
|  | 23.8－51.5 | 0 | 0 | 0 | 1 |  |  |
|  | 51.5 ≤ | 0 | 0 | 1 | 6 |  |  |
| BioPlex^®^  aβ_2_GPI IgM | < 30.6 | 83 | 5 | 1 | 0 | 0.51 | 0.383－0.644 |
|  | 30.6－68.8 | 1 | 0 | 1 | 0 |  |  |
|  | 68.8－156.6 | 0 | 0 | 1 | 4 |  |  |
|  | 156.6 ≤ | 0 | 0 | 0 | 4 |  |  |
| MEBLux^TM^ test aβ_2_GPI IgM | | < 16.6 | 16.6－37.7 | 37.7－85.9 | 85.9 ≤ | kappa statistic | 95%CI |
| QUANTA Flash^®^  aβ_2_GPI IgM | < 14.7 | 84 | 2 | 0 | 0 | 0.62 | 0.485－0.756 |
|  | 14.7－32.7 | 4 | 0 | 0 | 0 |  |  |
|  | 32.7－73.9 | 0 | 1 | 2 | 0 |  |  |
|  | 73.9 ≤ | 0 | 0 | 2 | 5 |  |  |
| EliA^TM^  aβ_2_GPI IgM | < 11.0 | 83 | 2 | 0 | 0 | 0.51 | 0.379－0.645 |
|  | 11.0－23.8 | 5 | 0 | 1 | 1 |  |  |
|  | 23.8－51.5 | 0 | 0 | 1 | 0 |  |  |
|  | 51.5 ≤ | 0 | 1 | 2 | 4 |  |  |
| BioPlex^®^  aβ_2_GPI IgM | < 30.6 | 87 | 2 | 0 | 0 | 0.72 | 0.581－0.854 |
|  | 30.6－68.8 | 1 | 0 | 1 | 0 |  |  |
|  | 68.8－156.6 | 0 | 1 | 3 | 1 |  |  |
|  | 156.6 ≤ | 0 | 0 | 0 | 4 |  |  |
| QUANTA Flash^®^ aβ_2_GPI IgM | | < 14.7 | 14.7－32.7 | 32.7－73.9 | 73.9 ≤ | kappa statistic | 95%CI |
| EliA^TM^  aβ_2_GPI IgM | < 11.0 | 83 | 2 | 0 | 0 | 0.69 | 0.555－0.832 |
|  | 11.0－23.8 | 3 | 2 | 1 | 1 |  |  |
|  | 23.8－51.5 | 0 | 0 | 1 | 0 |  |  |
|  | 51.5 ≤ | 0 | 0 | 1 | 6 |  |  |
| BioPlex^®^  aβ_2_GPI IgM | < 30.6 | 86 | 3 | 0 | 0 | 0.69 | 0.562－0.828 |
|  | 30.6－68.8 | 0 | 1 | 1 | 0 |  |  |
|  | 68.8－156.6 | 0 | 0 | 2 | 3 |  |  |
|  | 156.6 ≤ | 0 | 0 | 0 | 4 |  |  |
| EliA^TM^ aβ_2_GPI IgM | | < 11.0 | 11.0－23.8 | 23.8－51.5 | 51.5 ≤ | kappa statistic | 95%CI |
| BioPlex^®^  aβ_2_GPI IgM | < 30.6 | 84 | 5 | 0 | 0 | 0.54 | 0.413－0.666 |
|  | 30.6－68.8 | 1 | 1 | 0 | 0 |  |  |
|  | 68.8－156.6 | 0 | 0 | 1 | 4 |  |  |
|  | 156.6 ≤ | 0 | 1 | 0 | 3 |  |  |

aCL, anti-cardiolipin antibody; aβ_2_GPI, anti-β_2_-glycoprotein I antibody; CI, confidence interval
