# Supplementary material for: A preliminary report on the feasibility of regression-based alignment of diagnostic thresholds for harmonized use of international classification criteria for antiphospholipid syndrome
Source: PLoS One. 2025 Jul 24;20(7):e0328229. doi: 10.1371/journal.pone.0328229 (PMC12289022; doi:10.1371/journal.pone.0328229)
Supplement: S3 Table — aCL, anti-cardiolipin antibodies; aβ2GPI, anti-β2-glycoprotein I antibodies; CI, confidence interval. (DOCX) [file pone.0328229.s003.docx]

**S3 Table.** **Concordance of semi-quantitative classification across all assays for IgG isotypes, using thresholds predicted by the specificity-based method.**

| MESACUP^TM^-2 test aCL IgG | | < 8.6 | 8.6－10.1 | 10.1－16.3 | 16.3 ≤ | kappa statistic | 95%CI |
| --- | --- | --- | --- | --- | --- | --- | --- |
| QUANTA Lite^®^  aCL IgG | < 10.9 | 59 | 1 | 3 | 5 | 0.66 | 0.500－0.820 |
|  | 10.9－20.4 | 1 | 0 | 0 | 3 |  |  |
|  | 20.4－58.4 | 0 | 0 | 1 | 1 |  |  |
|  | 58.4 ≤ | 1 | 1 | 1 | 23 |  |  |
| QUANTA Flash^®^  aCL IgG | < 19.8 | 55 | 1 | 2 | 0 | 0.49 | 0.374－0.613 |
|  | 19.8－34.0 | 5 | 1 | 2 | 9 |  |  |
|  | 34.0－36.8 | 0 | 0 | 1 | 10 |  |  |
|  | 36.8 ≤ | 1 | 0 | 0 | 13 |  |  |
| EliA^TM^  aCL IgG | < 20.5 | 56 | 2 | 4 | 11 | 0.47 | 0.325－0.620 |
|  | 20.5－21.5 | 1 | 0 | 0 | 0 |  |  |
|  | 21.5－37.5 | 3 | 0 | 1 | 4 |  |  |
|  | 37.5 ≤ | 1 | 0 | 0 | 17 |  |  |
| BioPlex^®^  aCL IgG | < 4.5 | 50 | 1 | 2 | 5 | 0.53 | 0.378－0.676 |
|  | 4.5－15.1 | 7 | 0 | 1 | 1 |  |  |
|  | 15.1－32.3 | 2 | 0 | 0 | 2 |  |  |
|  | 32.3 ≤ | 2 | 1 | 2 | 24 |  |  |
| QUANTA Lite^®^ aCL IgG | | < 10.9 | 10.9－20.4 | 20.4－58.4 | 58.4 ≤ | kappa statistic | 95%CI |
| QUANTA Flash^®^  aCL IgG | < 19.8 | 56 | 8 | 2 | 2 | 0.48 | 0.361－0.604 |
|  | 19.8－34.0 | 0 | 3 | 1 | 0 |  |  |
|  | 34.0－36.8 | 1 | 1 | 0 | 0 |  |  |
|  | 36.8 ≤ | 1 | 5 | 8 | 12 |  |  |
| EliA^TM^  aCL IgG | < 20.5 | 54 | 12 | 4 | 3 | 0.33 | 0.217－0.452 |
|  | 20.5－21.5 | 0 | 1 | 0 | 0 |  |  |
|  | 21.5－37.5 | 3 | 3 | 0 | 2 |  |  |
|  | 37.5 ≤ | 1 | 1 | 7 | 9 |  |  |
| BioPlex^®^  aCL IgG | < 4.5 | 46 | 8 | 2 | 2 | 0.35 | 0.231－0.476 |
|  | 4.5－15.1 | 7 | 2 | 0 | 0 |  |  |
|  | 15.1－32.3 | 2 | 1 | 1 | 0 |  |  |
|  | 32.3 ≤ | 3 | 6 | 8 | 12 |  |  |
| QUANTA Flash^®^ aCL IgG | | < 19.8 | 19.8－34.0 | 34.0－36.8 | 36.8 ≤ | kappa statistic | 95%CI |
| EliA^TM^  aCL IgG | < 20.5 | 62 | 3 | 1 | 7 | 0.54 | 0.388－0.689 |
|  | 20.5－21.5 | 1 | 0 | 0 | 0 |  |  |
|  | 21.5－37.5 | 4 | 0 | 1 | 3 |  |  |
|  | 37.5 ≤ | 1 | 1 | 0 | 16 |  |  |
| BioPlex^®^  aCL IgG | < 4.5 | 57 | 0 | 0 | 1 | 0.68 | 0.528－0.826 |
|  | 4.5－15.1 | 7 | 2 | 0 | 0 |  |  |
|  | 15.1－32.3 | 2 | 1 | 0 | 1 |  |  |
|  | 32.3 ≤ | 2 | 1 | 2 | 24 |  |  |
| EliA^TM^ aCL IgG | | < 20.5 | 20.5－21.5 | 21.5－37.5 | 37.5 ≤ | kappa statistic | 95%CI |
| BioPlex^®^  aCL IgG | < 4.5 | 54 | 1 | 2 | 1 | 0.42 | 0.288－0.559 |
|  | 4.5－15.1 | 8 | 0 | 1 | 0 |  |  |
|  | 15.1－32.3 | 3 | 0 | 0 | 1 |  |  |
|  | 32.3 ≤ | 8 | 0 | 5 | 16 |  |  |

| QUANTA Lite^®^ aβ_2_GPI IgG | | < 4.0 | 4.0－17.3 | 17.3－37.1 | 37.1 ≤ | kappa statistic | 95%CI |
| --- | --- | --- | --- | --- | --- | --- | --- |
| MEBLux^TM^ test  aβ_2_GPI IgG | < 0.3 | 59 | 0 | 1 | 1 | 0.51 | 0.378－0.637 |
|  | 0.3－0.6 | 2 | 0 | 0 | 0 |  |  |
|  | 0.6－1.0 | 2 | 2 | 0 | 0 |  |  |
|  | 1.0 ≤ | 2 | 10 | 7 | 14 |  |  |
| QUANTA Flash^®^  aβ_2_GPI IgG | < 13.1 | 51 | 0 | 1 | 1 | 0.43 | 0.301－0.551 |
|  | 13.1－39.0 | 12 | 1 | 0 | 0 |  |  |
|  | 39.0－80.9 | 1 | 4 | 0 | 0 |  |  |
|  | 80.9 ≤ | 1 | 7 | 7 | 14 |  |  |
| EliA^TM^  aβ_2_GPI IgG | < 3.4 | 62 | 2 | 2 | 1 | 0.72 | 0.589－0.841 |
|  | 3.4－3.9 | 1 | 3 | 0 | 0 |  |  |
|  | 3.9－24.5 | 2 | 6 | 6 | 0 |  |  |
|  | 24.5 ≤ | 0 | 1 | 0 | 14 |  |  |
| BioPlex^®^  aβ_2_GPI IgG | < 14.1 | 58 | 0 | 1 | 1 | 0.51 | 0.383－0.642 |
|  | 14.1－18.4 | 3 | 1 | 0 | 0 |  |  |
|  | 18.4－40.1 | 3 | 1 | 0 | 0 |  |  |
|  | 40.1 ≤ | 1 | 10 | 7 | 14 |  |  |
| MEBLux^TM^ test aβ_2_GPI IgG | | < 0.3 | 0.3－0.6 | 0.6－1.0 | 1.0 ≤ | kappa statistic | 95%CI |
| QUANTA Flash^®^  aβ_2_GPI IgG | < 13.1 | 53 | 0 | 0 | 0 | 0.77 | 0.632－0.917 |
|  | 13.1－39.0 | 8 | 2 | 1 | 2 |  |  |
|  | 39.0－80.9 | 0 | 0 | 3 | 2 |  |  |
|  | 80.9 ≤ | 0 | 0 | 0 | 29 |  |  |
| EliA^TM^  aβ_2_GPI IgG | < 3.4 | 58 | 2 | 3 | 4 | 0.50 | 0.362－0.629 |
|  | 3.4－3.9 | 1 | 0 | 1 | 2 |  |  |
|  | 3.9－24.5 | 2 | 0 | 0 | 12 |  |  |
|  | 24.5 ≤ | 0 | 0 | 0 | 15 |  |  |
| BioPlex^®^  aβ_2_GPI IgG | < 14.1 | 60 | 0 | 0 | 0 | 0.89 | 0.725－1.000 |
|  | 14.1－18.4 | 0 | 2 | 2 | 0 |  |  |
|  | 18.4－40.1 | 1 | 0 | 1 | 2 |  |  |
|  | 40.1 ≤ | 0 | 0 | 1 | 31 |  |  |
| QUANTA Flash^®^ aβ_2_GPI IgG | | < 13.1 | 13.1－39.0 | 39.0－80.9 | 80.9 ≤ | kappa statistic | 95%CI |
| EliA^TM^  aβ_2_GPI IgG | < 3.4 | 51 | 12 | 2 | 2 | 0.44 | 0.319－0.561 |
|  | 3.4－3.9 | 1 | 0 | 2 | 1 |  |  |
|  | 3.9－24.5 | 1 | 1 | 1 | 11 |  |  |
|  | 24.5 ≤ | 0 | 0 | 0 | 15 |  |  |
| BioPlex^®^  aβ_2_GPI IgG | < 14.1 | 53 | 7 | 0 | 0 | 0.76 | 0.618－0.901 |
|  | 14.1－18.4 | 0 | 3 | 1 | 0 |  |  |
|  | 18.4－40.1 | 0 | 3 | 1 | 0 |  |  |
|  | 40.1 ≤ | 0 | 0 | 3 | 29 |  |  |
| EliA^TM^ aβ_2_GPI IgG | | < 3.4 | 3.4－3.9 | 3.9－24.5 | 24.5 ≤ | kappa statistic | 95%CI |
| BioPlex^®^  aβ_2_GPI IgG | < 14.1 | 57 | 1 | 2 | 0 | 0.50 | 0.371－0.634 |
|  | 14.1－18.4 | 3 | 1 | 0 | 0 |  |  |
|  | 18.4－40.1 | 4 | 0 | 0 | 0 |  |  |
|  | 40.1 ≤ | 3 | 2 | 12 | 15 |  |  |

aCL, anti-cardiolipin antibody; aβ_2_GPI, anti-β_2_-glycoprotein I antibody; CI, confidence interval
