# Supplementary material for: A preliminary report on the feasibility of regression-based alignment of diagnostic thresholds for harmonized use of international classification criteria for antiphospholipid syndrome
Source: PLoS One. 2025 Jul 24;20(7):e0328229. doi: 10.1371/journal.pone.0328229 (PMC12289022; doi:10.1371/journal.pone.0328229)
Supplement: S4 Table — aCL, anti-cardiolipin antibodies; aβ2GPI, anti-β2-glycoprotein I antibodies; CI, confidence interval. (DOCX) [file pone.0328229.s004.docx]

**S4 Table. Concordance of semi-quantitative classification across all assays for IgM isotypes, using thresholds predicted by the specificity-based method.**

| MESACUP^TM^-2 test aCL IgM | | < 5.1 | 5.1－8.9 | 8.9－10.4 | 10.4 ≤ | kappa statistic | 95%CI |
| --- | --- | --- | --- | --- | --- | --- | --- |
| QUANTA Lite^®^  aCL IgM | < 17.9 | 73 | 6 | 3 | 0 | 0.61 | 0.472－0.748 |
|  | 17.9－23.9 | 1 | 3 | 1 | 1 |  |  |
|  | 23.9－25.3 | 1 | 0 | 0 | 0 |  |  |
|  | 25.3 ≤ | 1 | 0 | 0 | 10 |  |  |
| QUANTA Flash^®^  aCL IgM | < 15.0 | 72 | 9 | 4 | 0 | 0.24 | 0.117－0.366 |
|  | 15.0－31.9 | 2 | 0 | 0 | 4 |  |  |
|  | 31.9－681 | 1 | 0 | 0 | 5 |  |  |
|  | 681 ≤ | 1 | 0 | 0 | 2 |  |  |
| EliA^TM^  aCL IgM | < 25.0 | 73 | 9 | 4 | 3 | 0.36 | 0.236－0.488 |
|  | 25.0－30.5 | 2 | 0 | 0 | 0 |  |  |
|  | 30.5－43.5 | 1 | 0 | 0 | 1 |  |  |
|  | 43.5 ≤ | 0 | 0 | 0 | 7 |  |  |
| BioPlex^®^  aCL IgM | < 25.1 | 73 | 9 | 4 | 2 | 0.20 | 0.084－0.311 |
|  | 25.1－45.8 | 1 | 0 | 0 | 1 |  |  |
|  | 45.8－3277 | 1 | 0 | 0 | 7 |  |  |
|  | 3277 ≤ | 1 | 0 | 0 | 1 |  |  |
| QUANTA Lite^®^ aCL IgM | | < 17.9 | 17.9－23.9 | 23.9－25.3 | 25.3 ≤ | kappa statistic | 95%CI |
| QUANTA Flash^®^  aCL IgM | < 15.0 | 78 | 5 | 1 | 1 | 0.36 | 0.231－0.483 |
|  | 15.0－31.9 | 2 | 1 | 0 | 3 |  |  |
|  | 31.9－681 | 1 | 0 | 0 | 5 |  |  |
|  | 681 ≤ | 1 | 0 | 0 | 2 |  |  |
| EliA^TM^  aCL IgM | < 25.0 | 80 | 4 | 1 | 4 | 0.46 | 0.323－0.605 |
|  | 25.0－30.5 | 2 | 0 | 0 | 0 |  |  |
|  | 30.5－43.5 | 0 | 1 | 0 | 1 |  |  |
|  | 43.5 ≤ | 0 | 1 | 0 | 6 |  |  |
| BioPlex^®^  aCL IgM | < 25.1 | 79 | 5 | 1 | 3 | 0.31 | 0.193－0.421 |
|  | 25.1－45.8 | 1 | 1 | 0 | 0 |  |  |
|  | 45.8－3277 | 1 | 0 | 0 | 7 |  |  |
|  | 3277 ≤ | 1 | 0 | 0 | 1 |  |  |
| QUANTA Flash^®^ aCL IgM | | < 15.0 | 15.0－31.9 | 31.9－681.3 | 681 ≤ | kappa statistic | 95%CI |
| EliA^TM^  aCL IgM | < 25.0 | 82 | 2 | 3 | 2 | 0.29 | 0.163－0.414 |
|  | 25.0－30.5 | 2 | 0 | 0 | 0 |  |  |
|  | 30.5－43.5 | 1 | 1 | 0 | 0 |  |  |
|  | 43.5 ≤ | 0 | 3 | 3 | 1 |  |  |
| BioPlex^®^  aCL IgM | < 25.1 | 85 | 3 | 0 | 0 | 0.80 | 0.660－0.933 |
|  | 25.1－45.8 | 0 | 2 | 0 | 0 |  |  |
|  | 45.8－3277 | 0 | 1 | 6 | 1 |  |  |
|  | 3277 ≤ | 0 | 0 | 0 | 2 |  |  |
| EliA^TM^ aCL IgM | | < 25.0 | 25.0－30.5 | 30.5－43.5 | 43.5 ≤ | kappa statistic | 95%CI |
| BioPlex^®^  aCL IgM | < 25.1 | 83 | 2 | 2 | 1 | 0.25 | 0.124－0.376 |
|  | 25.1－45.8 | 1 | 0 | 0 | 1 |  |  |
|  | 45.8－3277 | 4 | 0 | 0 | 4 |  |  |
|  | 3277 ≤ | 1 | 0 | 0 | 1 |  |  |

| QUANTA Lite^®^ aβ_2_GPI IgM | | < 17.1 | 17.1－37.9 | 37.9－70.7 | 70.7 ≤ | kappa statistic | 95%CI |
| --- | --- | --- | --- | --- | --- | --- | --- |
| MEBLux^TM^ test  aβ_2_GPI IgM | < 14.7 | 80 | 5 | 0 | 0 | 0.49 | 0.364－0.619 |
|  | 14.7－33.3 | 2 | 2 | 2 | 0 |  |  |
|  | 33.3－1488 | 0 | 0 | 1 | 6 |  |  |
|  | 1488 ≤ | 0 | 0 | 0 | 2 |  |  |
| QUANTA Flash^®^  aβ_2_GPI IgM | < 11.0 | 78 | 5 | 0 | 0 | 0.55 | 0.416－0.679 |
|  | 11.0－35.4 | 4 | 2 | 1 | 0 |  |  |
|  | 35.4－205.7 | 0 | 0 | 2 | 4 |  |  |
|  | 205.7 ≤ | 0 | 0 | 0 | 4 |  |  |
| EliA^TM^  aβ_2_GPI IgM | < 3.7 | 72 | 4 | 0 | 0 | 0.48 | 0.345－0.605 |
|  | 3.7－11.5 | 9 | 2 | 0 | 0 |  |  |
|  | 11.5－92.0 | 1 | 1 | 2 | 3 |  |  |
|  | 92.0 ≤ | 0 | 0 | 1 | 5 |  |  |
| BioPlex^®^  aβ_2_GPI IgM | < 25.5 | 81 | 6 | 0 | 0 | 0.50 | 0.378－0.624 |
|  | 25.5－45.7 | 1 | 1 | 1 | 0 |  |  |
|  | 45.7－2590 | 0 | 0 | 2 | 6 |  |  |
|  | 2590 ≤ | 0 | 0 | 0 | 2 |  |  |
| MEBLux^TM^ test aβ_2_GPI IgM | | < 14.7 | 14.7－33.3 | 33.3－1488 | 1488 ≤ | kappa statistic | 95%CI |
| QUANTA Flash^®^  aβ_2_GPI IgM | < 11.0 | 80 | 3 | 0 | 0 | 0.61 | 0.479－0.750 |
|  | 11.0－35.4 | 5 | 2 | 0 | 0 |  |  |
|  | 35.4－205.7 | 0 | 1 | 5 | 0 |  |  |
|  | 205.7 ≤ | 0 | 0 | 2 | 2 |  |  |
| EliA^TM^  aβ_2_GPI IgM | < 3.7 | 74 | 2 | 0 | 0 | 0.47 | 0.343－0.602 |
|  | 3.7－11.5 | 9 | 2 | 0 | 0 |  |  |
|  | 11.5－92.0 | 2 | 1 | 4 | 0 |  |  |
|  | 92.0 ≤ | 0 | 1 | 3 | 2 |  |  |
| BioPlex^®^  aβ_2_GPI IgM | < 25.5 | 84 | 3 | 0 | 0 | 0.80 | 0.662－0.943 |
|  | 25.5－45.7 | 1 | 2 | 0 | 0 |  |  |
|  | 45.7－2590 | 0 | 1 | 7 | 0 |  |  |
|  | 2590 ≤ | 0 | 0 | 0 | 2 |  |  |
| QUANTA Flash^®^ aβ_2_GPI IgM | | < 11.0 | 11.0－35.4 | 35.4－205.7 | 205.7 ≤ | kappa statistic | 95%CI |
| EliA^TM^  aβ_2_GPI IgM | < 3.7 | 76 | 0 | 0 | 0 | 0.66 | 0.531－0.793 |
|  | 3.7－11.5 | 7 | 4 | 0 | 0 |  |  |
|  | 11.5－92.0 | 0 | 3 | 4 | 0 |  |  |
|  | 92.0 ≤ | 0 | 0 | 2 | 4 |  |  |
| BioPlex^®^  aβ_2_GPI IgM | < 25.5 | 83 | 4 | 0 | 0 | 0.78 | 0.644－0.912 |
|  | 25.5－45.7 | 0 | 3 | 0 | 0 |  |  |
|  | 45.7－2590 | 0 | 0 | 6 | 2 |  |  |
|  | 2590 ≤ | 0 | 0 | 0 | 2 |  |  |
| EliA^TM^ aβ_2_GPI IgM | | < 3.7 | 3.7－11.5 | 11.5－92.0 | 92.0 ≤ | kappa statistic | 95%CI |
| BioPlex^®^  aβ_2_GPI IgM | < 25.5 | 76 | 10 | 1 | 0 | 0.48 | 0.359－0.606 |
|  | 25.5－45.7 | 0 | 1 | 2 | 0 |  |  |
|  | 45.7－2590 | 0 | 0 | 4 | 4 |  |  |
|  | 2590 ≤ | 0 | 0 | 0 | 2 |  |  |

aCL, anti-cardiolipin antibody; aβ_2_GPI, anti-β_2_-glycoprotein I antibody; CI, confidence interval
